# Corrigendum

**DOI:** 10.1002/1348-9585.12372

**Published:** 2022-11-24

In Sasaki et al.,^1^ the wrong data are included in some columns of Tables 3 and 4 due to misalignment.

**TABLE 3:** The odds ratio for suicidal ideation among Japanese employees in the COVID‐19 pandemic (August 2020) by using multinominal logistic regression analysis (*N* = 12 249)

	Persistent suicidal ideation		Newly developed suicidal ideation in the COVID‐19 pandemic		Persistent suicidal ideation		Newly developed suicidal ideation in the COVID‐19 pandemic	
	Crude		Crude		Adjusted^a^		Adjusted^a^	
	OR	95% CI	OR	95% CI	OR	95% CI	OR	95% CI
Employment status								
Permanent	1.00	—	1.00	—	1.00	—	1.00	—
Temporary	1.37	1.20–1.57	1.25	1.00–1.55	1.36	1.16–1.59	1.10	0.85–1.42
Gender								
Male	1.00	—	1.00	—	1.00	—	1.00	—
Female	1.14	1.01–1.30	1.44	1.17–1.76	0.95	0.82–1.11	1.24	0.98–1.57
Age (years)								
50–65	1.00	—	1.00		1.00	—	1.00	
40–49	1.63	1.37–1.94	2.09	1.54–2.85	1.52	1.26–1.82	1.94	1.42–2.66
30–39	1.75	1.45–2.10	2.78	2.04–3.79	1.73	1.42–2.10	2.86	2.08–3.93
20–29	2.39	1.98–2.89	3.44	2.50–4.73	1.99	1.62–2.46	3.40	2.41–4.79
Marital status								
Married	1.00	—	1.00	—	1.00	—	1.00	—
Single	2.19	1.93–2.50	1.53	1.25–1.87	1.82	1.58–2.10	1.11	0.89–1.38
Education attainment								
Four‐year university or more	1.00	—	1.00		1.00	—	1.00	
Vocational/College	1.07	0.91–1.25	1.20	0.93–1.54	1.06	0.89–1.27	1.18	0.90–1.55
High school	1.07	0.91–1.24	1.20	0.94–1.54	1.03	0.87–1.23	1.30	0.99–1.70
Company size (employees)								
≥1000	1.00	—	1.00	—	1.00	—	1.00	—
300–999	1.11	0.90–1.37	1.43	1.05–1.96	1.05	0.84–1.31	1.32	0.95–1.82
50–299	1.17	0.98–1.40	1.09	0.82–1.46	1.10	0.91–1.33	0.98	0.73–1.33
<50 employees	1.21	1.02–1.43	1.09	0.83–1.43	1.15	0.96–1.39	0.95	0.71–1.28
Industry								
Public office	1.00	—	1.00	—	1.00	—	1.00	—
Forestry and mining	1.79	0.73–4.35	2.76	0.79–9.62	1.51	0.60–3.79	2.68	0.75–9.59
Construction	1.31	0.91–1.89	1.51	0.82–2.78	1.29	0.87–1.89	1.58	0.84–2.95
Manufacturing	1.15	0.85–1.54	1.26	0.76–2.07	1.10	0.81–1.49	1.24	0.75–2.06
Electricity, gas, and water supply	1.98	1.19–3.29	1.87	0.78–4.47	1.87	1.10–3.17	1.84	0.76–4.48
Information and technology	1.42	0.99–2.04	1.22	0.64–2.31	1.27	0.87–1.84	1.18	0.62–2.26
Transportation	1.53	1.06–2.22	1.66	0.89–3.10	1.49	1.01–2.20	1.79	0.95–3.39
Retail and wholesale	1.31	0.96–1.80	1.38	0.81–2.36	1.13	0.81–1.59	1.28	0.73–2.23
Finance and insurance	0.99	0.66–1.49	1.07	0.54–2.12	1.02	0.67–1.56	1.10	0.55–2.20
Real estate	1.26	0.74–2.15	1.23	0.49–3.10	1.19	0.68–2.08	1.26	0.49–3.22
Drinking, eating, and hotel	1.79	1.22–2.63	2.72	1.50–4.91	1.25	0.83–1.90	2.08	1.11–3.90
Medical and welfare	1.22	0.89–1.66	1.84	1.11–3.04	1.11	0.79–1.54	1.66	0.98–2.82
Education	1.17	0.79–1.72	1.18	0.61–2.28	1.11	0.74–1.66	1.31	0.67–2.58
Others	1.29	0.96–1.73	1.54	0.94–2.52	1.05	0.77–1.44	1.42	0.85–2.37
History of psychiatric disease								
No	1.00	—	1.00	—	1.00	—	1.00	—
Yes	5.19	4.51–5.97	5.31	4.28–6.58	4.99	4.33–5.76	5.36	4.31–6.67

Abbreviation: OR, odds ratio.

^a^ Adjusted for sex, age category, marital status, education, company size, industries, and history of psychiatric disease.

**Table 4:** The odds ratio for suicidal ideation among Japanese employees in the COVID‐19 pandemic (August 2020) stratified by gender by using multinominal logistic regression analysis (*N* = 12 249)

	Total *N*	Persistent suicidal ideation		Newly developed suicidal ideation in the COVID‐19 pandemic		Persistent suicidal ideation		Newly developed suicidal ideation in the COVID‐19 pandemic	
		Crude		Crude		Adjusted^a^		Adjusted^a^	
		OR	95% CI	OR	95% CI	OR	95% CI	OR	95% CI
Men (*n* = 7095)									
Employment status									
Permanent	6213	1.00	—	1.00	—	1.00	—	1.00	—
Temporary	882	1.51	1.20–1.90	1.20	0.80–1.82	1.15	0.89–1.49	1.02	0.65–1.60
Age (years)									
50–65	1591	1.00	—	1.00	—	1.00	—	1.00	—
40–49	2018	1.70	1.34–2.17	1.52	0.98–2.34	1.59	1.24–2.05	1.45	0.93–2.26
30–39	1591	1.96	1.52–2.51	2.67	1.77–4.02	1.93	1.49–2.51	2.82	1.85–4.30
20–29	1066	2.97	2.30–3.83	3.11	2.01–4.82	2.33	1.76–3.09	3.15	1.96–5.09
Marital status									
Married	4444	1.00	—	1.00	—	1.00	—	1.00	—
Single	2651	2.41	2.03–2.86	1.48	1.11–1.98	1.86	1.53–2.26	1.07	0.77–1.48
Education attainment									
Four‐year university or more	4590	1.00	—	1.00	—	1.00	—	1.00	—
Vocational/College	980	1.14	0.89–1.45	1.10	0.72–1.67	1.08	0.83–1.40	1.11	0.72–1.71
Highschool	1525	1.04	0.84–1.29	1.08	0.76–1.53	1.04	0.83–1.31	1.20	0.83–1.76
Company size (employees)									
≥1000	2244	1.00	—	1.00	—	1.00	—	1.00	—
300–999	1077	1.23	0.94–1.62	1.40	0.92–2.13	1.17	0.88–1.55	1.31	0.86–2.02
50–299	1813	1.28	1.01–1.61	1.25	0.86–1.81	1.16	0.91–1.48	1.11	0.76–1.64
<50	1961	1.28	1.02–1.60	0.78	0.52–1.18	1.21	0.94–1.55	0.73	0.47–1.13
Industry									
Public office	690	1.00	—	1.00	—	1.00	—	1.00	—
Forestry and mining	35	1.44	0.42–4.90	3.25	0.70–15.04	1.20	0.34–4.18	3.85	0.81–18.28
Construction	439	1.25	0.79–1.99	1.74	0.81–3.75	1.25	0.77–2.04	2.12	0.96–4.68
Manufacturing	1754	1.26	0.89–1.79	1.30	0.69–2.44	1.22	0.85–1.75	1.34	0.70–2.53
Electricity, gas, and water supply	132	1.78	0.95–3.36	2.16	0.75–6.16	1.61	0.83–3.12	2.30	0.79–6.74
Information and technology	524	1.70	1.12–2.58	1.50	0.70–3.21	1.50	0.98–2.32	1.57	0.72–3.42
Transportation	437	1.39	0.88–2.20	2.29	1.11–4.74	1.36	0.85–2.19	2.67	1.27–5.64
Retail and wholesale	634	1.29	0.85–1.96	1.20	0.56–2.57	1.24	0.80–1.93	1.46	0.67–3.19
Finance and insurance	300	0.77	0.42–1.41	0.87	0.31–2.46	0.90	0.48–1.66	1.09	0.38–3.13
Real estate	136	1.30	0.65–2.58	1.20	0.34–4.27	1.42	0.70–2.92	1.72	0.47–6.29
Drinking, eating, and hotel	151	2.17	1.23–3.84	2.32	0.87–6.22	1.55	0.84–2.85	2.50	0.89–6.99
Medical and welfare	546	1.25	0.80–1.93	1.91	0.93–3.9	1.12	0.70–1.77	1.95	0.93–4.10
Education	303	1.51	0.92–2.48	1.65	0.70–3.9	1.44	0.85–2.42	1.92	0.79–4.66
Others	1014	1.36	0.93–1.98	1.51	0.78–2.94	1.13	0.76–1.69	1.66	0.84–3.32
History of psychiatric disease									
No	6141	1.00	—	1.00	—	1.00	—	1.00	—
Yes	954	4.73	3.92–5.71	5.27	3.90–7.12	4.77	3.93–5.80	5.59	4.10–7.61
Women (*n* = 5154)									
Employment status									
Permanent	2659	1.00	—	1.00	—	1.00	—	1.00	—
Temporary	2495	1.27	1.05–1.54	1.03	0.77–1.37	1.51	1.21–1.88	1.11	0.80–1.54
Age (years)									
50–65	1619	1.00	—	1.00	—	1.00	—	1.00	—
40–49	1550	1.53	1.18–1.98	2.80	1.79–4.41	1.38	1.06–1.81	2.58	1.63–4.08
30–39	1069	1.51	1.14–2.00	2.93	1.82–4.73	1.46	1.08–1.97	2.94	1.80–4.81
20–29	916	1.80	1.36–2.39	3.75	2.33–6.01	1.63	1.19–2.23	3.82	2.30–6.35
Marital status									
Married	2620	1.00	—	1.00	—	1.00	—	1.00	—
Single	2534	1.92	1.57–2.33	1.45	1.09–1.94	1.81	1.46–2.24	1.19	0.87–1.63
Education attainment									
Four‐year university or more	1963	1.00	—	1.00	—	1.00	—	1.00	—
Vocational/College	1804	0.95	0.76–1.19	1.05	0.75–1.48	1.02	0.80–1.31	1.25	0.86–1.80
Highschool	1387	1.02	0.81–1.30	1.18	0.83–1.69	1.00	0.76–1.31	1.42	0.96–2.10
Company size (employees)									
≥1000	1053	1.00	—	1.00	—	1.00	—	1.00	—
300–999	682	0.91	0.64–1.28	1.38	0.86–2.24	0.91	0.64–1.31	1.30	0.79–2.13
50–299	1315	0.98	0.74–1.30	0.86	0.55–1.36	1.02	0.76–1.39	0.83	0.52–1.33
<50	2104	1.03	0.80–1.33	1.17	0.79–1.74	1.08	0.81–1.43	1.13	0.74–1.72
Industry									
Public office	247	1.00	—	1.00	—	1.00	—	1.00	—
Forestry and mining	18	2.22	0.59—8.37	1.95	0.23–—16.68	1.95	0.47–8.18	1.75	0.19–15.76
Construction	231	1.31	0.71–2.42	1.10	0.41–2.99	1.24	0.65–2.36	1.04	0.37–2.92
Manufacturing	557	0.91	0.53–1.57	1.22	0.53–2.78	0.82	0.46–1.46	1.13	0.49–2.65
Electricity, gas, and water supply	50	2.40	1.02–5.62	1.40	0.29–6.83	2.37	0.97–5.79	1.40	0.28–7.06
Information and technology	157	0.80	0.38–1.72	0.77	0.23–2.60	0.70	0.32–1.53	0.64	0.19–2.22
Transportation	127	1.98	1.03–3.81	0.52	0.11–2.49	1.89	0.95–3.77	0.50	0.10–2.46
Retail and wholesale	699	1.15	0.69–1.92	1.17	0.52–2.62	0.94	0.54–1.63	1.01	0.44–2.33
Finance and insurance	284	1.04	0.57–1.91	0.98	0.37–2.59	1.02	0.54–1.92	0.95	0.35–2.55
Real estate	86	1.11	0.47–2.61	1.09	0.28–4.22	0.87	0.35–2.14	0.83	0.21–3.33
Drinking, eating, and hotel	298	1.35	0.75–2.40	2.10	0.90–4.88	0.99	0.53–1.85	1.58	0.65–3.85
Medical and welfare	1007	1.00	0.61–1.66	1.33	0.62–2.88	0.99	0.58–1.68	1.29	0.58–2.87
Education	312	0.77	0.41–1.44	0.67	0.24–1.88	0.76	0.39–1.47	0.82	0.28–2.35
Others	1081	1.06	0.64–1.73	1.21	0.56–2.62	0.90	0.53–1.53	1.11	0.50–2.46
History of psychiatric disease									
No	4472	1.00	—	1.00	—	1.00	—	1.00	—
Yes	682	5.88	4.76–7.26	5.47	4.03–7.44	5.39	4.35–6.70	5.10	3.73–6.97

Abbreviation: OR, odds ratio.

The corrected Tables 3 and 4 are given below.

The authors apologize for this error.
